# Statistics for approximate gene clusters

**DOI:** 10.1186/1471-2105-14-S15-S14

**Published:** 2013-12-13

**Authors:** Katharina Jahn, Sascha Winter, Jens Stoye, Sebastian Böcker

**Affiliations:** 1 Genome Informatics, Faculty of Technology, Bielefeld University, 33615 Bielefeld, Germany; 2 Institute for Bioinformatics, Center for Biotechnology (CeBiTec), Bielefeld University, 33615 Bielefeld, Germany; 3 Chair for Bioinformatics, Friedrich-Schiller-University Jena, Germany

## Abstract

**Background:**

Genes occurring co-localized in multiple genomes can be strong indicators for either functional constraints on the genome organization or remnant ancestral gene order. The computational detection of these patterns, which are usually referred to as gene clusters, has become increasingly sensitive over the past decade. The most powerful approaches allow for various types of imperfect cluster conservation: Cluster locations may be internally rearranged. The individual cluster locations may contain only a subset of the cluster genes and may be disrupted by uninvolved genes. Moreover cluster locations may not at all occur in some or even most of the studied genomes. The detection of such low quality clusters increases the risk of mistaking faint patterns that occur merely by chance for genuine findings. Therefore, it is crucial to estimate the significance of computational gene cluster predictions and discriminate between true conservation and coincidental clustering.

**Results:**

In this paper, we present an efficient and accurate approach to estimate the significance of gene cluster predictions under the approximate common intervals model. Given a single gene cluster prediction, we calculate the probability to observe it with the same or a higher degree of conservation under the null hypothesis of random gene order, and add a correction factor to account for multiple testing. Our approach considers all parameters that define the quality of gene cluster conservation: the number of genomes in which the cluster occurs, the number of involved genes, the degree of conservation in the different genomes, as well as the frequency of the clustered genes within each genome. We apply our approach to evaluate gene cluster predictions in a large set of well annotated genomes.

## Background

Gene order-based analysis of whole genomes has become an important field in comparative genomics. It is well known that genomes evolve, not only at the level of nucleotide sequence, but also by means of large-scale rearrangements, such as inversions and transpositions, as well as changes of the gene content. Focusing on this higher-level structure, genomes are usually modeled as sequences of integers with genes belonging to the same gene family represented by the same integer. If no selective pressure was acting on whole genome evolution, gene order and gene content would randomize over time. In reality, particularly in bacterial genomes, we observe low overall conservation of gene order between species, contrasted by a small number of well-conserved segments. These are often referred to as *gene clusters*. Such local aberrations from genome randomization provide evidence for various biological phenomena and are of high interest in functional and evolutionary genomics [1-9].

When comparing a large number of genomes, the identification of these structures can be a challenging task since conservation patterns may be highly variable across species. Due to micro-rearrangements, gene order can also vary between cluster occurrences. Gene insertions and losses, or mis-annotations may lead to cluster occurrences interrupted by genes that do not belong to the cluster, or containing only a subset of the clustered genes. Moreover, a gene cluster may be present only in a subset of the genomes under study. (A gene cluster displaying all these features, except for mis-annotations, is shown in Additional File 1) Taking these effects into account in different ways, several models of gene clusters and algorithms for their detection have been suggested [10-17].

However, it may as well be the case that seemingly conserved patterns occur merely by chance. To estimate the likeliness of such events, appropriate statistics are needed to quantify the probability of finding gene clusters by chance. Such statistics have been developed for some gene cluster models, in particular  r*-windows *[18-20], *segmental homologies *(k -clumps) [21], and max-gap clusters [22-24]. Other methods solve the problem of assigning significance to predicted gene clusters in an ad-hoc manner, including C-Hunter [25], OrthoCluster [26], MCMuSeC [16], CYNTENATOR [27], and i-ADHoRe [28]. In this paper we consider the gene cluster model of approximate common intervals [15]. This can be easily applied to a variety of use cases, as it offers a combination of parameter flexibility and efficiency of computation, even for very large data sets. The variant *reference gene clusters *[17] proves especially useful. We provide a statistical test for evaluating gene cluster predictions against the null hypothesis of random gene order. For our background model, we consider, for each genome  G, a random string  S of the same length where each character (gene) has a probability proportional to its frequency in  G. For multi-chromosomal genomes, or in cases where the (unfinished) genome sequence consists of multiple contigs, we do the same for each chromosome/contig individually. We then estimate p-values, that is, the probabilities of gene clusters of the observed quality or higher being found in the random genomes by chance.

Since the random genomes are drawn independently, we can proceed as follows: For each genome, we compute the likelihood of a gene cluster occurring in the corresponding random genome. These are the individual p-values for each genome. Next, we demonstrate how to combine p-values from individual genomes into one p-value for the gene cluster. The problem of multiple testing is then considered by applying a false discovery rate control to minimize this effect. Finally, we demonstrate that there is excellent concurrence between our calculated p-values and empirically determined p-values and that the method is able to recognize known gene clusters from large genomic data sets.

## Methods

### Preliminaries

We model a genome as a *string *over a finite alphabet Σ={1, . . . , σ} of gene family ids, such that genes belonging to the same homology family are represented by the same integer. In the following, we use the terms "genome" and "string" and, also, "gene" and "character" interchangeably. Given a string S, we denote its *length *by  and refer to its  ith character as S[i], 1≤i≤|S|. A character c∈Σ is said to *occur *at position  i in  if S[i]=c. A substring of  S from position  a to  b is denoted S[a, b] for 1≤a≤b≤|S|. To capture the character content of S[a, b] regardless of sequential arrangement and multiple character occurrences, we define the *character set *of S[a,b] as

$$CS\left(S\left[a,\;\;b\right]\right):=\{S\left[i\right]:a\leq i\leq b\text{\}}\subseteq \sum .$$

The corresponding index interval [a,b] is termed a *location *of C⊆Σ if and only if C=CS(S[a, b]). An interval [a, b] is *left-maximal *if a=1 or S[a-1]∉CS(S[a, b]); it is *right-maximal *if b=|S| or $S\left[b+\text{1}\right]\notin CS\left(S\left[a,\;b\right]\right);$ and it is *maximal *if it is both left- and right-maximal.

Given k≥2 strings $S_{\text{1}},\;.\;.\;.\;S_{k},$ k maximal intervals written as  k-tuple $\left(\left[a_{\text{1}},b_{\text{1}}\right],\;.\;.\;.\;,\;\left[a_{k},b_{k}\right]\right)$ are called *common intervals *in $S_{\text{1}},\;.\;.\;.\;S_{k}$ if and only if

$$CS(S_{1}\left[a_{1},b_{1}]\right)=...=CS(S_{k}\left[a_{k},b_{k}]\right)=:C.$$

Given that $S_{\text{1}},\;.\;.\;.,\;S_{k}$ are genomes, the above character set  C corresponds to a gene cluster with perfectly conserved gene content.

In order to model gene clusters with incomplete conservation patterns, we quantify the differences in the gene content of their approximate gene cluster occurrences via their *symmetric set distance*. This measure defines the distance between two finite sets  and $C^{\prime }$ as the cardinality of their *symmetric difference*:

$$D\left(C,C^{\prime }\right):=|C\backslash C^{\prime }|+|C^{\prime }\backslash C|=|C\cup C^{\prime }\left|-\right|C\cap C^{\prime }|.$$

This constitutes a metric and therefore meets all intuitive notions of a distance measure, such as validity of the triangle inequality. In the context of gene clusters, it corresponds to a simple summation over the genes deleted and the genes inserted into a cluster occurrence. Like earlier character set based gene cluster models [29], it disregards recurrences of genes within the same cluster occurrence.

Based on this distance notion, we extend the concept of character set locations towards approximate conservation: Given an integer δ≥0, we define an interval [a,b] in a string  as a -*location *of character set C, if and only if, D(C,CS(S[a,b]))≤δ, and C∩CS(S[a,b])≠∅.

Let $S_{\text{1}},\;.\;.\;.,\;S_{k}$ be genomes over a gene alphabet . Let s≥2 be the minimum cluster size, δ≥0 a distance threshold and $k^{\prime }$ a *quorum *parameter with $\text{2}\leq k^{\prime }\leq k$. A *reference gene cluster *for parameters s,δ and $k^{\prime }$ is a set of genes C⊆Σ with |C|≥s such that  has an exact occurrence in one of the genomes and δ-locations in at least $k^{\prime }-1$ other genomes. In other words, there exist i,a,b such that $C=CS\left(S_{i}\left[a,b\right]\right)$, and J⊆{1, . . . ,k}-{i} with $|J|\;\geq k^{\prime }-1$ such that each $S_{j}$ has a -location of  for all .

In [17] we studied the following problem: Given genomes $S_{\text{1}},\;.\;.\;.,\;S_{k}$ and parameters $s,\delta ,k^{\prime },$ find all reference gene clusters C⊆Σ in $S_{\text{1}},\;.\;.\;.\;,S_{k},$ and for each reference gene cluster, output all its optimal δ-locations. (A δ-location is not optimal if it is a subinterval of a δ-location that has a smaller distance to C.) We introduced an efficient algorithm that runs in $O\left(k^{\text{2}}n^{\text{2}}\delta^{\text{2}}+k^{\text{2}}n^{\text{2}}\right)$ time using $O\left(kn^{\text{2}}\right)$ space, where  n is the length of the largest genome [17]. The algorithm is exact, meaning that it is guaranteed to find all reference gene clusters and their optimal occurrences as specified by the search parameters.

The above definitions do not take into account multi-chromosomal genomes, or genomes that were not completely assembled and still consist of several contigs. However, it is simple to extend these definitions, as well as the remainder of this paper, to the multi-chromosomal or multi-contig case. For example, we may assume that the different chromosomes/contigs of one genome are concatenated in a single string, separated by symbols $∉Σ. We can then assume that neither a gene cluster nor an interval is allowed to contain the character . Further details will be omitted, aside from saying that some of the complexity bounds mentioned below actually improve for multi-chromosomal and multi-contig genomes.

### Significance of a gene cluster for one genome

In this section we estimate the probability of a fixed gene cluster C⊆Σ having a  δ-location in a random genome  of length , i. e. the p-value of finding an occurrence of  in genome S:

(1) p-value=ℙ(Shas a δ-location of C)

In the following we assume that δ≤|C|-2 holds. Otherwise the p-value is equal to one whenever C∩CS(S)≠∅, and zero otherwise. Let p(L,d)=p(L,d,C) be the probability that a random occurrence of length  has a symmetric set distance *exactly * to C. Let q(L,δ)=q(L,δ,C) be the probability that it has a symmetric set distance of *at most *δ. Note that p(L,d) and q(L,δ) depend on the cluster C; in the following, our notations omit this dependency for the sake of readability. Then, $q\left(L,\delta \right)=\sum_{d=0}^{\delta }p\left(L,d\right)$. To simplify our computation, we assume that occurrence probabilities are independent for all intervals [*a*, *b*] where 1≤a≤b≤n. Clearly, this assumption is not correct: Let  A be the event that interval [i,j] forms a δ-location of C, and let  be the event that interval $\left[i,j+\text{1}\right]$ forms a -location of C. The fact that the intervals share all positions but one creates a number of non-trivial dependencies. In case S[j+1]∈CS(S[i, j]), the two events are either both true or both false. When the distance between CS(S[i, j]) and  is smaller than δ, p(B|A)=1, and vice versa. Such dependencies apply not only to [i,j] and [i,j+1], but to all intersecting interval pairs. However, we will show later on that the p-values computed under this assumption are very close to the true p-values, for any realistic choices of parameters. We estimate the p-value for a single genome as

(2) $$\begin{array}{l} \mathbb{P}\left(S\;\text{has a }\delta \text{-}\text{locations of }C\right)\approx 1-\underset{a\leq b}{\prod }\left(1-q\left(b-a+1,\delta \right)\right)\\ =1-\underset{L=1,...,n}{\prod }{\left(1-q\left(L,\delta \right)\right)}^{n-L+1}. \end{array}$$

To minimize the effects of rounding error accumulation, we instead compute

(3) $$\mathbb{P}\left(S\;\text{has a }\delta \text{-}\text{location of }C\right)\approx 1-\text{exp}\left(\underset{L=1,...,n}{\sum }\left(n-L+1\right)\cdot \text{log}\;\left(1-q\left(L,\;\delta \right)\right)\right)$$

which can be calculated with high accuracy, using mathematical library functions for f(x):=exp(x)-1 and g(x):=log(x+1).

#### Exact computation using dynamic programming

We need to compute p(L,d), the probability that the character set of a random interval of length  L has a symmetric set distance  d to a given (fixed) gene cluster C, for all  and d≤δ. Let $S_{L}$ be a random string of length , and let ℙ(c) denote the probability of character c∈Σ for any position of the random string. For a sub-alphabet $\Sigma^{\prime }\subseteq \Sigma$, set $\mathbb{P}\left(\Sigma \prime \right)\;:=\underset{c\in \Sigma \prime }{\sum }\mathbb{P}\left(c\right).$ The distance  d between  C and $CS\;\left(S_{L}\right)$ can be partitioned as $d=d_{-}+d_{+}$: Here, $d_{-}$ is the number of characters from  that are *missing *in $S_{L}$, and $d_{+}$ is the number of *additional *characters in $CS\left(S_{L}\right).$ Consequently, we can partition the positions in $S_{L}$ into two types: those positions containing characters from C, and those positions containing characters from $\bar{C}:=\sum \;-\;C.$ Assume that  positions of  are occupied by characters from >, 0≤l≤L, and that L-l positions are occupied by characters from $\bar{C}$. We calculate

(4) $$\begin{array}{c} p\left(L,\;d\right)=\overset{d}{\underset{d\text{\_}=0}{\sum }}\mathbb{P}\left(\;|C\backslash CS\left(S_{L}\right)|\;=\;d\text{\_}\wedge |CS\left(S_{L}\right)\backslash C|\;=d-d\text{\_}\right)\\ =\overset{d}{\underset{d\text{\_}=0}{\sum }}\overset{L}{\underset{l=0}{\sum }}\mathbb{P}\left(|\;\left\{i|S_{L}\left[i\right]\in C,1\leq i\leq L\right\}|\;=l\wedge |C\backslash \;CS\left(S_{L}\right)|\;=d\text{\_}\wedge |CS\left(S_{L}\right)\backslash C|=d-d\text{\_}\right)\\ =\overset{L}{\underset{l=0}{\sum }}\overset{d}{\underset{d\text{\_}=0}{\sum }}p_{0}\left(l,L\right).\;p^{-}\left(l,d\text{\_}\right).\;p^{+}\left(L-l,\;d-d\text{\_}\right) \end{array}$$

where $p_{0}\left(l,L\right)$ is the probability of drawing a random string of length  L with  l characters from  and L-l characters from $\bar{C};p^{-}\left(l,d\text{\_}\right)$ is the probability that, for a random string $S_{l^{}}$ of length  over the alphabet , $CS\left(S_{l}\right)$ is missing $d_{-}$ characters from ; and $p^{+}\left(L-l,d-d\text{\_}\right)$ is the probability that, for a random string $S_{l^{\prime }}$ of length $l^{\prime }=L-l$ over the alphabet $\bar{C},CS\left(S_{l^{\prime }}\right)$ contains $d_{+}=d-d\text{\_}$ different characters. If all these values are known, we can compute the desired probability using (4) in time O(dL). In practice, we found that $p^{-}\left(l,d_{-}\right)$ in (4) decreases rapidly with increasing . To this end, we can stop the summation, as well as the computation of $p^{-}\left(l,d_{-}\right)$ and $p^{+}\left(L-l,\;d_{+}\right)$, as soon as $\overset{d}{\underset{d\text{\_}=0}{\sum }}p_{0}\left(l,L\right).\;p^{-}\left(l,d\text{\_}\right).\;p^{+}\left(L-l,\;d_{+}\right)$ no longer contributes to the sum.

Computing $p_{0}\left(l,L\right)$ is straightforward, using the binomial distribution: One can see that

$$p_{0}\left(l,L\right)=\left(\begin{array}{c} L\\ l \end{array}\right)\cdot \mathbb{P}\;{\left(C\right)}^{l}\cdot \mathbb{P}\;{\left(\bar{C}\right)}^{L-l}$$

holds. It remains to be shown how to compute $p^{-}\left(l,d_{-}\right)$ for missing characters and $p^{+}\left(L-l,d_{+}\right)$ for additional characters.

#### Missing characters

We first demonstrate how to compute $p^{-}\left(l,d_{-}\right)$, the probability that a random string $S_{l}$ of length  over the alphabet  is missing $d_{-}$ characters from C. Let $h:=\;|C|-d_{-}$ be the number of *hits *from $CS\left(S_{l}\right)$ in C. For readability, we base our computation on the number of hits h. The order of the characters in the random string is not relevant, so we simply check whether a certain character from  has been generated. The statistical equivalent is rolling dice. We assume, for simplicity, that C:={1,…,Z}. Probabilities of the characters in  are conditional probabilities of the same characters in . We define

$$p\left[z,l,h\right]:=\mathbb{P}\left(h\;\text{different outcomes for }l\;\text{rolls for }{\#}^{\prime }s\;1,...,z\right).$$

So, p[z,l,h] is the probability that, by throwing  dice with numbers 1,…,z, exactly  different numbers have been rolled. In the following, we not only iterate over the number of rolled dice and different outcomes, but also over the numbers that can be rolled. In cases where only numbers 1,…,z out of 1,…,Z can be rolled, we can calculate the conditional probability of each outcome with the recurrence:

(5) $$\begin{array}{c} p[z,l,h]=\mathbb{P}\text{(no }z\text{ rolled with }l\text{ dice)}\cdot p[z-1,l,h]\\ +\sum_{\ell =1}^{l}\mathbb{P}(\ell \text{ times }z\text{ rolled with }l\text{ dice})\cdot p[z-1,l-\ell ,h-1]) \end{array}$$

We initialize p[z,0,0]=1,p[z,0,h]=0,p[z,l,0]=0, and p[0,l,h]=0 for all  and all l,h>0. The two missing probabilities are computed using a binomial distribution. In the end, $p^{-}(l,d_{-})=p[Z,l,h]$ is the probability that in a random string of length  over alphabet , exactly  different characters from  have been generated. Computation takes $O\left(|C|hl^{\text{2}}\right)$ time and O(hl) space, as only the values for z=Z need to be stored.

#### Additional characters

The recurrence introduced in equation (5), can also be used to compute $p^{+}\left(l,d_{+}\right)$. We need to set $h:=d_{+}$, and exchange the roles of  and $\bar{C}.$ Unfortunately the latter modification has a strong impact on the practical runtime, due to the linear dependence now being on the much larger $\left|\bar{C}\right|$ (compared to the rather small |C| for $p^{-}\left(l,d_{-}\right)$). However, we can mitigate this by pooling genes based on the frequency of their occurrences in the original genome. The genes within each pool have the same occurrence probability in the random genome and need not be distinguished in our calculations. It is sufficient to know for each pool how many of its genes originate from , and $\bar{C}$ respectively.

Let  f be the number of different occurrence frequencies observed in the original genome, and let $F_{\text{1}},\;.\;.\;.\;,F_{f}$ denote the corresponding gene pools. Given a fixed gene cluster , we denote by $F_{z}^{\bar{C}}$ the subset of pool $F_{z},\text{ 1 }\leq z\leq f,$ that consists only of genes from $\bar{C}.$ We then modify recurrence (5) as follows:

$$\begin{array}{l} p[z,l,h]=\mathbb{P}(\text{no gene of }F_{z}^{\bar{C}}\;\text{rolled with }l\text{ dice})\cdot p[z-1,l,h]\\ \;+\sum_{\ell =1}^{l}\sum_{h'=1}^{\min(h,|F_{z}^{\bar{C}}|)}(\mathbb{P}\;(\ell \text{ genes of }F_{z}^{\bar{C}},\;h'\text{ differnet ones},\text{ rolled with }l\text{ dice}).\;p\;[z-1,l-\ell ,h-h']) \end{array}$$

The initializations are the same as for the previous recurrence. We can use the binomial distribution to compute the value of  ℙ (no gene of $F_{z}^{\bar{C}}$ rolled with  l dice) and the same type of recurrence as in equation (5) to compute the second probability,  ℙ( genes of $F_{z}^{\bar{C}}$, $h^{\prime }$ different ones, rolled with  l dice). In the end, $p^{+}\left(l,d_{+}\right)=p\left[f,l,h\right]$ is the probability that in a random string of length  l over alphabet $\bar{C},$ exactly  h different characters from $\bar{C}$ have been generated.

Due to the second summation, the asymptotic time complexity of the recurrence becomes $O\left(fh^{\text{2}}l^{\text{2}}\right)$. We observe that, in practice f, the number of different gene occurrence frequencies is very small compared to $\bar{C}$ and is typically in the size range of large gene clusters. Also, the vast majority of genes occur only once in a genome, with pool sizes dropping quickly for larger occurrence frequencies. Since $h^{\prime }$ is bounded, not only by *h *but also by $|F_{f}^{\bar{C}}|,$ the quadratic dependence on  h is unlikely to be relevant in practice.

Next, we show how the values of  ℙ( genes of $F_{z}^{\bar{C}}$, $h^{\prime }$ different ones, rolled with  dice) can efficiently be computed. Ideally, we would like to precompute these values once for every genome, providing constant-time lookup during computation of p-values for the different gene clusters. At first sight, these probabilities seem to be specific for each cluster, as the gene pools need to be restricted to their complement $\bar{C}$. However, we know that all genes in a pool have the same occurrence probability, therefore it is sufficient to compute the above values for all residual pools, after removing a certain number - not a certain set - of genes. For small pools, which are in the majority, not much extra work is required to do this for all possible residual sizes. For large pools exact computations may, in practice, be too costly. In this case, we suggest working with pools based on the complete alphabet . Due to their size, the removal of a few elements has little influence on the conditional probabilities of the remaining elements.

In practice, we use a faster, but less exact approach: We replace the more accurate estimation of $p^{+}\left(l,d_{+}\right)$ with a much faster preprocessing that leads to almost identical results. To this end, we compute a global $P^{+}\left(l,d_{+}\right)$ for the complete alphabet, $\bar{C}:=\;\Sigma ,$ during preprocessing. This is achieved using recurrence (5). We then assume that, for any cluster  with additional character probabilities $p^{+}\left(l,d_{+}\right)$, we have $p^{+}\text{(}l,d_{+}\text{)}\approx P^{+}\left(l,d_{+}\right)$. In doing so, we ignore the fact that the gene cluster  C removes some of the genes from the pool of potential additional genes. Clearly, this computation can be carried out very quickly, as we have to compute $P^{+}\left(l,d_{+}\right)$ only once for each genome. Depending on the distribution of occurrence probabilities, the above approximation can distort the results significantly. We account for this by setting $p^{+}\left(l,d_{+}\right)\approx {{\left(\text{1}-\mathbb{P}\left(C\right)\right)}^{d}}^{+}\cdot P^{+}\left(l,d_{+}\right)$, thereby taking into account the occurrence probabilities of the genes in C.

### Significance of a gene cluster in multiple genomes

Thus far, we have concentrated on the probability of observing gene cluster occurrences in a single genome. To estimate the significance of observing a gene cluster in multiple genomes, we need to combine the individual probabilities into a single p-value. This gives the probability of observing a gene cluster C, at least as well conserved in the randomized genomes as in the original genome set.

We begin by formalizing the notion of "at least as well conserved". Consider the case where a  δ-location of  C is detected in all genomes $S_{\text{1}},\;.\;.\;.\;,S_{k}$. To simplify notation, we assume that $S_{\text{1}},\;.\;.\;.\;,S_{k}$ are the remaining genomes after removing the reference genome, the one from which we took the interval to generate C. Clearly no p-value estimation is necessary for this genome, and it can be omitted from the following calculations. Let $d=\left(d_{\text{1}},\;.\;.\;.\;,d_{k}\right)$ be a distance vector, such that $d_{i}$ is the distance between  C and its best approximate occurrence in genome $S_{i},\text{ 1}\leq i\leq k$. We denote by **d**_obs_ the distance vector observed for  C and the original genomes. To make different distance configurations comparable, we need to define a linearorder of all possible distance vectors. We chose an ordering based on the total distance, $\sum_{i=1}^{k}d_{i}.$ We denote by $d_{\text{obs}}$ the sum distance of **d**_obs_. To exclude configurations with δ-locations in fewer genomes than observed in the original data, we further require that each individual distance in the vector is at most δ. To calculate the probability of observing any distance vector that satisfies the above constraints, we define the following recurrence:

(7) $$M\left[i,d\right]=\overset{\text{min}\left(d,\delta \right)}{\underset{d^{\prime }=0}{\sum }}\left(\mathbb{P}\left(\text{best }\delta \text{-}\text{location in }S_{i\;}\text{has distance }d^{\prime }to\;C\right)\cdot M\left[i-1,d-d^{\prime }\right]\right).$$

The base cases are M[0, 0]=1, and M[0,d]=0 for d>0.  ℙ(best δ-locations in $S_{i}$ has distance $d^{\prime }$ to ) equals  ℙ( has a $d^{\prime }$-location in $S_{i}$) −  ℙ( C has a $\left(d^{\prime }-\text{1}\right)$-location in $S_{i}$). These probabilities can be computed with equation (2). Summing over all M[k,d], $0\leq d\leq d_{\text{obs}}$, gives the desired p-value. i. e. the probability that  is at least as well conserved in the randomized genomes as in the original dataset. This computation takes time $O\left(k^{\text{2}}\delta^{\text{2}}\right)$, as  i and  d are bounded by  and $d_{\text{obs}}$, respectively, and we have $d_{\text{obs}}\leq k\cdot \delta$.

When a gene cluster is observed only in a subset of the studied genomes, it becomes tricky to define the meaning of "at least as well conserved": Is a gene cluster better conserved if it occurs in many genomes at a low quality, or in fewer genomes but at higher quality? We suggest that the latter should be given preference. Otherwise, there is the risk of systematically preferring gene clusters that occur in a large number of genomes, yet only incompletely in the form of one or two genes, over clusters that are very well conserved but only in a small number of genomes. We believe that the latter are the more interesting cases. Therefore we say a gene cluster  with -locations in $k^{\prime }$ out of  genomes and sum distance $d_{\text{obs}}$ is conserved at the same or better quality in the randomized genomes, if it has δ-locations in at least $k^{\prime }$ of them, and the best $k^{\prime }$δ-locations (from  k different genomes) have a sum distance of at most $d_{\text{obs}}$ to . Unfortunately the recurrence used in equation (7) cannot be extended to compute the corresponding probabilities. We need to track the sum of the $k^{\prime }$ smallest distances below , after processing the first i≤k genomes. This value cannot be computed in a simple recursive manner, as there is no optimal substructure underlying the problem: The sum, after processing  genomes, depends not only on the sum before the genome was added and the distance for the new genome, but also on the previous number of distances below  and the values of these distances. Only with all of this information is it possible to decide whether or not the distance encountered for the new genome needs to be added to the sum. In the absence of an efficient dynamic programming approach, we need to sum probabilities over all ${k^{\delta }}^{+\text{1}}$ distance vectors. This becomes infeasible for larger .

In order to avoid exponential running time, we studied a simpler approach where we use a fixed distance threshold $\delta^{\prime }$ for all genomes. This can be either the original threshold , or the largest entry in **d**_obs_, i. e. the largest of the distances between  and its best δ-location in each genome.

As a consequence, we do not need to sum over pairwise distances in the recurrence, but over the number of genomes that contain a $\delta^{\prime }$-location. Let $p_{j}$ be the likelihood of a $\delta^{\prime }$-location in genome $S_{j},$ computed using equation (2). The likelihood of having $\delta^{\prime }$-locations in exactly  i out of j genomes $S_{\text{1}},\;.\;.\;.\;,S_{j},\text{ 1}\leq i\leq j\leq k$, can be computed using the recurrence

(8) $$Q\left[i,j\right]\;=\;\left(\text{1}-p_{j}\right)\cdot Q\left[i,j-\text{1}\right]\;+p_{j}\cdot Q\;\left[i-\text{1},j-\text{1}\right].$$

The base cases are $Q\left[0,\;\text{1}\right]=\text{1}-p_{\text{1}},\;Q\left[\text{1},\;\text{1}\right]=p_{\text{1}}$, and Q[i,j]=0 for all i>j. The likelihood of finding at least $k^{\prime }$$\delta^{\prime }$-locations in  k genomes is then the sum over all Q[i,k] with $k^{\prime }\leq i\leq k.$ Computation requires $O\left(k^{\text{2}}\right)$ time. This method will be referred to as "global distance bound".

Unfortunately, the above approach has the following problem: Consider two (otherwise identical) gene clusters, both found in three genomes. The first cluster is found with distances 0, 1 and 4 in the three genomes; the second cluster with distances 3, 4 and 4. Common sense tells us that the first gene cluster is more significant, because it is less likely to occur by chance. However, the approach described above will come up with identical likelihoods, as, in both cases we have $\delta^{\prime }=\text{4}$. In fact, for the first cluster it may be beneficial to exclude the last occurrence, as this may lead to a smaller p-value; we omit the details.

To ensure that gene clusters of this type are evaluated differently while keeping the computational complexity reasonable, we resort to the following simplification: Recall that $\delta^{\prime }\leq \delta$ is the maximum distance of the cluster to any occurrence. For genomes where we do not detect a -location, we use the single-genome p-value with distance threshold $\delta^{\prime }$; for genomes other than the reference genome that contain a δ-location (and, hence, a $\delta^{\prime }$-location), we use the single-genome p-value with distance threshold given by the distance of the detected interval in this genome. In the above example, for the first cluster, we use distance threshold 0 for the first genome, 1 for the second genome, and 4 for the third genome; for the second cluster we use distance threshold 3 for the first genome and 4 for the remaining two. This method will be referred to as "individual distance bounds".

### False discovery rate control

Since we are testing significance not only for a single gene cluster, but for the complete set of gene cluster predictions reported by a search extending over all possible reference intervals, we need to adjust our p-values accordingly. We use false discovery rate (FDR) control [30] to counteract the problem of multiple testing. In detail, we sort all the clusters by their p-value and multiply the p-value  p of any gene cluster with the number of possible reference intervals in all  k genomes divided by the index  i in the sorted cluster list:

(9) $$p_{i}^{FDR}=p_{i}.\frac{\overset{k}{\underset{j=1}{\sum }}\frac{n_{j}.\left(n_{j}+1\right)}{2}}{i},$$

where $n_{\text{1}},\;.\;.\;.\;,n_{k}$ are the genome lengths. This is a conservative estimation and comes at the cost of increasing the probability of producing false negatives. That is, gene clusters that should be regarded as significant may be declared non-significant after the FDR correction. In addition, equation (9) actually overestimates the number of gene clusters tested as we do not take into consideration certain gene clusters that appear multiple times in a genome (and should be tested only once). We do not use the more powerful Šidák correction [31], as independence of the different tests cannot be guaranteed. FDR-corrected p-values can be larger than one, which solely indicates that this correction is conservative.

### Evaluation of p-value accuracy for a single genome

We now evaluate the accuracy of the p-value estimation we introduced above for a single genome. Recall that we use two simplifications to keep this task computationally feasible. First, we assume that the intervals within a genome do not overlap, in order to gain statistical independence of the probabilities q(L,δ) in equation (2). Second, we employ a heuristic approach to deal with additional characters in cluster occurrences. To show that our calculations are still sufficiently accurate, we compare our estimated p-values with p-values derived empirically from large-scale simulations of random genomes.

Two simulation studies were performed, one with biological data and one with simulated data. To reduce simulation time, we performed the first study on four, somewhat small, bacterial genomes with just 600 to 850 genes each, namely *Buchnera aphidicola APS*, *Ureaplasma urealyticum*, *Mycoplasma pneumoniae*, and *Borrelia burgdorferi*. We downloaded these genomes from the NCBI database [32] and assigned homology families, using GHOSTFAM[33] with standard parameters. Ten billion random sequences were then generated, with the same length and character frequencies as the original genomes.

Our reference gene cluster detection algorithm [17] was applied to the four original genomes, with three different parameter settings (δ=1,s=4), (δ=3,s=6), and (δ=5,s=9). The quorum parameter was set to $k^{\prime }=\text{2}$ in each case. This search returned 459 gene clusters. We computed for each gene cluster , the p-value of each occurrence, excluding the reference interval. This was done with equation (2), by setting the threshold to $\delta^{\prime }$, the symmetric set distance between  and the character set of the occurrence. Next, the p-value for a genome with randomized gene order containing a $\delta^{\prime }$-location of  was empirically determined. To this end, all random genomes corresponding to the genome where the occurrence was observed were searched for intervals with a symmetric set distance of, at most, $\delta^{\prime }$ to  C. The number of genomes with at least one such occurrence was divided by the total number of tested genomes. This gives an empirical estimate of the true p-value. When no occurrences were found, we omitted the pair from further analysis, as this shows the empirical p-value to be out of the scope of the current evaluation. This resulted in the omission of 277 gene clusters. The maximum p-value calculated for any such omitted pair was 9.23 · 10^−11^. Therefore all of the omitted values fall into the 95% Agresti-Coull confidence interval [34] of finding no occurrences, that has an upper bound of 4.64 · 10^−10^. (Note that when searching for gene clusters, what matters in p-value estimation is the *order of magnitude*. For example, p-values 2.7 · 10^−380^ and 5.4 · 10^−380^ differ by a factor of two; still, we would regard a gene cluster with either of these p-values as *highly *significant.)

**Figure 1:**
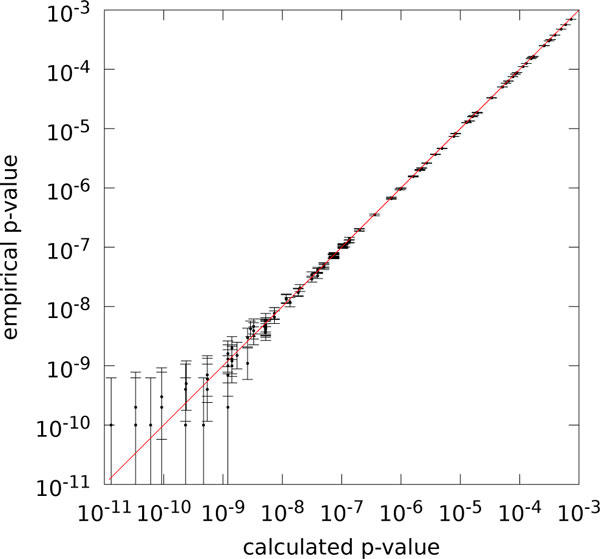
**Comparison of empirical and calculated p-values for bacterial genomes**. Comparison of empirical and calculated p-values for gene clusters from four bacterial genomes (log-log plot, N=182). Pearson correlation of log-log-pairs is $r=+0.\text{9976775 (}r^{\text{2}}=0.\text{99536}0\text{4)}$, coefficient of determination of log-log-pairs is $R^{\text{2}}=0.\text{9951661}$ (identity in red). Error bars visualize the 95% Agresti-Coull confidence interval.

For the remaining 182 gene clusters, we have plotted the calculated p-values against the empirical p-values in Figure 1. Unless our independence assumption is severely violated, this should result in points close to the main diagonal. Note that both the calculated p-values and the empirical p-values can deviate from the true p-value; this estimate becomes highly inaccurate, particularly for very small empirical p-values, as only few of the billions of genomes contain an occurrence of the gene cluster.

Nevertheless, we find an excellent agreement between the calculated p-values and the empirical p-values. For numerical comparison of these values, we transform both into the log scale: Otherwise, almost all p-values are far too small to contribute to Pearson correlation or coefficient of determination. The Pearson correlation of the log-log pairs is $r=+0.\text{9976775 (}r^{\text{2}}=0.\text{99536}0\text{4)}$. As we want to use the calculated p-values as a predictor of the empirical p-values, we also computed the coefficient of determination

$$R^{2}=1-\frac{\underset{i}{\sum }{\left(y_{i}-x_{i}\right)}^{2}}{\underset{i}{\sum }{\left(y_{i}-\bar{y}\right)}^{2}}$$

where the $y_{i}$ are the observations (log empirical p-values), the $x_{i}$ are the predictions (log calculated p-values), and $\bar{y}$ is the mean of the observations. For the bacterial genomes, we achieve $R^{\text{2}}=0.\text{9951661}$. The observed deviations for small probabilities must be attributed to the fact that, for these reference gene clusters, ten billion random genomes are not enough to give a good estimate of the true value. It appears very likely that the empirical p-value is inaccurate, rather than the calculated p-value.

We argue that these results strongly indicate that our calculated p-values are very close to the true values; hence, although equation (2) does not take into account statistical dependencies, our calculations are still highly accurate.

The number of p-value pairs in the above study is relatively small and not sufficient to firmly conclude that our calculations are accurate. To obtain a greater degree of certainty, we performed a second study using random genomes. Here, random genomes of different sizes and with different character distributions were generated. In order to create random genomes with similar characteristics to true biological data, we studied the gene family size distribution in real genomes. (A complete list of the genomes can be found in Additional file 1). Gene family size appears to roughly follow a heavy-tailed distribution (Additional file 1). The Pareto distribution was therefore selected for simulating genomes. The probability density function is:

(10) $$f\left(x\right)=\frac{\alpha x_{m}^{\alpha }}{x^{\alpha +1}},\;\alpha ,k>0,\;x\geq x_{m}.$$

In the following calculations, we use $x_{m}=\text{1}$, so that each gene appears at least once. The bacterial genomes we use later on for our evaluation approximately follow a Pareto distribution with α=2.8 (Additional file 1).

For each random genome we uniformly draw its length n∈[1250, 1750]. To select the character content of the genome, we repeat the following: We choose the next character and draw the number of occurrences of this character in the random genome using the above Pareto distribution. We repeat this until all  n positions of the random genome are filled, discarding surplus copies of the last added character. In this manner, we generated ten genome contents.

To generate a random gene order, we could concatenate the genes and shuffle the resulting string. To speed up computations, we proceeded in a slightly different way: Instead of generating a random genome and then searching for reference gene clusters, we simply assume a gene cluster  to be present. To obtain useful p-values we combined different strategies to construct : (1) nine clusters are chosen with two to ten genes using the most commonly occurring genes; (2) nine clusters are chosen with two to ten genes using the rarest genes, usually occurring only once; (3) 82 clusters are chosen with two to ten genes, randomly selected. For each of these 100 gene clusters we randomly choose a maximum allowed distance δ∈[0,|C|-2]. As mentioned earlier, for higher values of  δ exact p-values can be easily computed, simply by testing whether a single gene from  is present in the genome.

We then proceeded as described above, comparing the calculated p-value to an empirical p-value obtained from one billion random draws. From the 10 · 100 = 1000 gene clusters tested, we omitted 249 p-value pairs where no occurrences were empirically observed. The maximum calculated p-value for any such omitted pair is 2.45 · 10^−9^, while the upper bound of the 95% Agresti-Coull confidence interval for observing no occurrence is 4.64 · 10^−9^. To further increase the accuracy of the empirical estimation of the 49 clusters that were found between one and ten times only, we did an additional nine billion random draws. For five clusters, we still observed less than ten occurrences; the largest calculated p-value among these clusters is 5.40 · 10^−10^, the upper bound of the 95% Agresti-Coull interval for observing nine occurrences is 1.74 · 10^−9^. As for these cases the empirically determined p-values are presumably inaccurate, we excluded them from our analysis.

**Figure 2:**
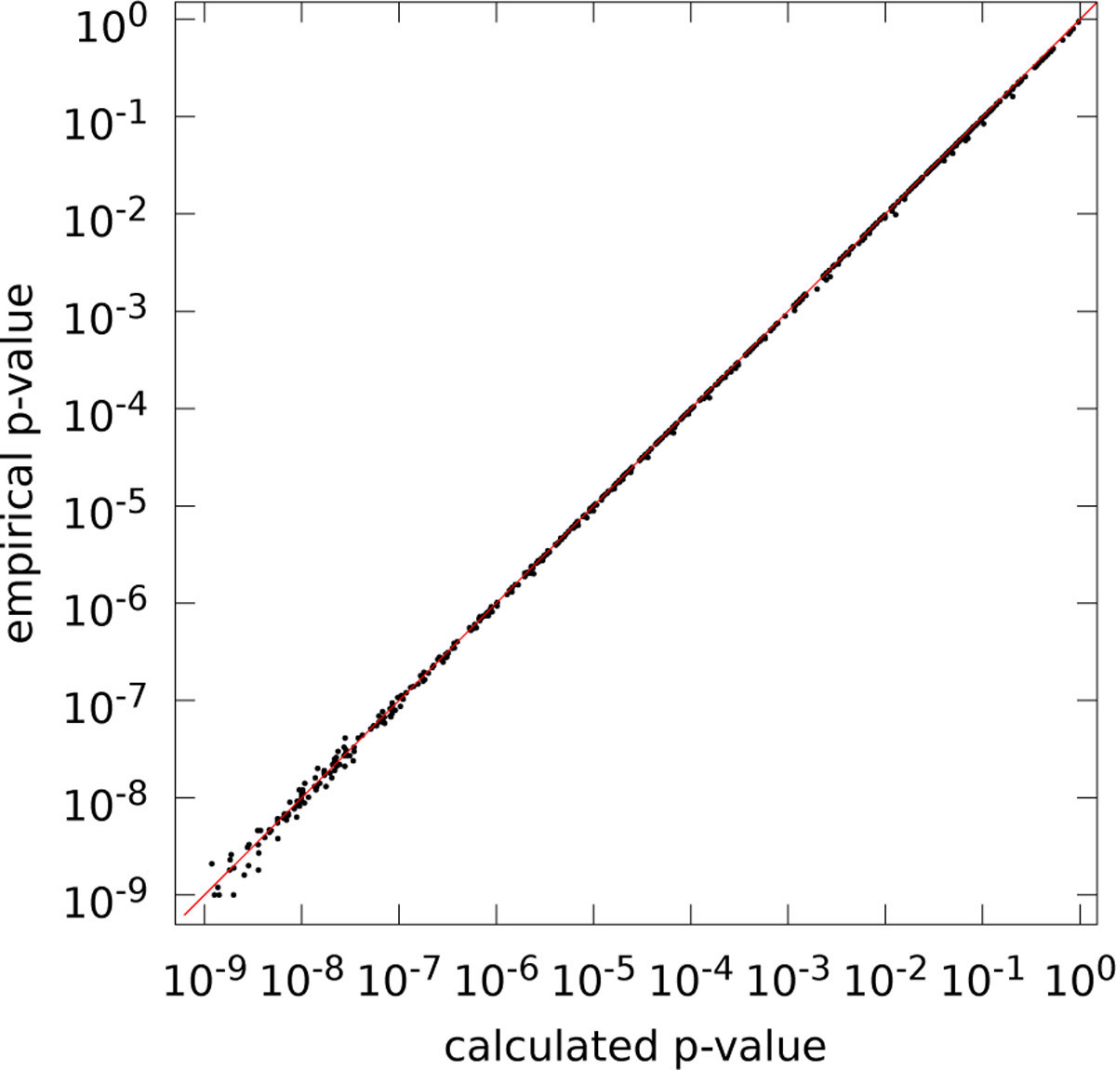
**Comparison of empirical and calculated p-values for simulated data**. Comparison of empirical and calculated p-values for random gene clusters in random genomes (log-log plot, N=746). Pearson correlation of log-log-pairs is $r=\;+0.\text{99989 (}r^{\text{2}}=\;0.\text{999783)}$, coefficient of determination of log-log-pairs is $R^{\text{2}}=0.\text{99975}$ (identity in red).

For the remaining 746 gene clusters, the calculated p-values vs. empirical p-values are plotted in Figure 2. Again, we see an excellent agreement between calculated and empirical p-values: Pearson correlation of the log-log pairs is $r=+0.\text{99989 (}r^{\text{2}}=0.\text{999783)}$, and the coefficient of determination of the log-log pairs is $R^{\text{2}}=0.\text{99975}$.

## Results

To evaluate our method, we used a dataset of 119 bacterial genomes from the RefSeq database [32]. A complete list of the genomes can be found in Additional File 1. This dataset has previously been used by Ling *et al*. [16] for the evaluation of the MCMuSeC software; we removed 14 plasmids. A partitioning of the genes of these genomes into homology families is available based on COG (Clusters of Orthologous Groups) numbers [35]. We deliberately ran our analysis on this set of well-described bacteria to facilitate evaluation of our cluster predictions based on the annotations provided in the database.

We searched for reference clusters with *Mycobacterium tuberculosis *CDC1551 (refSeq NC_002755) as the reference genome, which contains 4189 genes. We used five different combinations of  and  s, namely (δ=0,s=4), (δ=1,s=5), (δ=2,s=6), (δ=3,s=7) and (δ=5,s=8). The quorum parameter was set to $k^{\prime }=\text{1}0$ in each case. Finding the gene clusters took about 9.3 minutes on a laptop computer (run as a single thread on an Intel i5 M520 processor, 2.40 GHz, 8 GB RAM). For multiple genome p-value calculation, we applied the "individual distance bounds" method. Computing p-values for the resulting 582 gene clusters (including duplicates) required 1.2 minutes. The p-values were FDR-corrected for the 8, 775, 955 intervals in the *M. tuberculosis *genome. Gene cluster lists were merged and duplicate occurrences removed. For gene clusters where at least one occurrence, in one of the genomes, intersected, we report only the one with the smaller p-value. This resulted in 63 gene clusters. The best 20 are shown in Table 1; the complete list can be found in Additional File 1. The gene cluster with the best p-value (3.24 · 10^−1308^ after FDR correction) is found in 108 out of the 119 genomes; it contains nine genes with functional annotations linked to the 50S and 30S ribosomal subunits. By contrast, the second most significant gene cluster appears in 114 genomes and shows a much higher degree of conservation with regards to inserted and deleted genes. However, it contains just seven genes (functional annotation is also 50S/30S ribosomal subunits); its p-value is 6.61 · 10^−1252^. The cluster in position four of the list (NADH dehydrogenase) is only contained in 57 genomes. The p-value, of 2.68 · 10^−891^, is still very low as it contains nine genes and a low average distance of 1.4.

**Table 1:** Top 20 gene cluster predictions.

distance to ref.								
**ID**	**G**	**GN**	**min**	**max**	**avg**	**p-score**	**corr. p-score**	**description**
1	9	108	2	5	2.8	1314.43	1307.49	30S/50S ribosomal subunit
2	7	114	0	3	1.6	1258.12	1251.18	30S/50S, rpoA, infA
3	6	91	0	2	0.7	1031.47	1024.83	ATP synthase
4	9	57	0	5	1.4	896.31	890.57	NADH dehydrogenase
5	8	108	3	5	4.1	716.68	711.29	30S/50S ribosomal subunit
6	8	88	0	5	4.2	569.88	564.63	phosphate ABC transporter
7	8	93	0	5	4.1	486.80	481.67	infB, rfbA, nusA, hypothetical protein
8	8	79	3	5	4.6	367.33	362.27	putative/peptide ABC transporter
9	8	62	3	5	4.4	294.41	289.40	sugar ABC transporter
10	8	65	2	5	4.1	290.24	285.24	N-acetylmuramoyl, cell division
11	4	33	0	0	0.0	272.99	267.55	succinate dehydrogenase
12	8	51	3	5	4.9	221.73	216.79	pdhA/B/C
13	8	48	2	5	4.9	216.54	211.62	ATP-dependent (Clp) protease, trigger factor
14	8	58	0	5	4.2	216.12	211.20	50S L31, prfA, thrA/B/C, rho, hemK
15	8	50	4	5	4.9	213.61	208.70	hisA/C/F/H
16	6	32	0	2	1.7	200.11	194.80	dnaA/N, gyrA/B, recF
17	6	27	1	2	1.7	194.39	189.10	carA/B, pyrC/B/R
18	8	67	4	5	5.0	192.56	187.69	elongation factor Tu, G; 30S S7
19	8	29	4	5	4.5	190.62	185.75	sulfate ABC transporter
20	8	44	2	5	4.3	181.13	176.28	argB/C/D/G/H/F/R

The first 20 gene clusters found when searching *Mycobacterium tuberculosis *CDC1551 against 118 bacterial genomes. Clusters are sorted by p-values, computed using the "individual distance bounds" method. "G" is the number of different genes in the reference gene cluster; "GN" is the number of genomes where the reference gene cluster is found; "distance to ref." indicates the observed distances between the reference gene cluster and its occurrences. The "p-score" is the negative log_10_ of the p-value, before and after FDR correction.

We have also computed p-values using the "maximum distance bound" method, see Additional file 1. The best scoring cluster, in both cases, is part of the 30S/50S ribosomal subunit, with nine conserved genes. However, using the "maximum distance bound" method, the best scoring occurrence of this cluster is only found in 66 genomes, with a p-value of 1.19 · 10^−946^, while the occurrence in 108 genomes only receives a p-value of 1.43 · 10^−714^ due to its maximum distance of only five. The p-value for the 66 genomes occurrence using the "individual distance bounds" method is 2.37 · 10^−947^, while the 108 genomes occurrence receives a p-value of 3.24 · 10^−1308^.

None of the clusters in the above experiments have a corrected p-value anywhere close to 0.05, the typical threshold used to discriminate significant from non-significant observations. To get some insight into this "grey area" where no confident predictions can be made, we studied gene cluster predictions with a corrected p-value close to 0.05. We obtained these in three runs of our program, $\left(s=\text{3},\;\delta =\text{1},\;k^{\prime }=\text{9}\right)$, $\left(s=\text{4},\;\delta =\text{4},\;k^{\prime }=\text{8}\right)$ and $\left(s=\text{6},\;\delta =\text{7},\;k^{\prime }=\text{7}\right)$. For each setting we collected all predictions with a p-value > 0.05 (corrected p-score < -1.3), and also the same number of predictions with the biggest p-values < 0.05. The complete list of these 84 predictions can be found in Additional file 1. We compared the predictions with known E. coli operons that we obtained from the RegulonDB database [36]. As can be seen in Additional file 1 most of the 84 predictions above and below the threshold contain at least one operon. A more formal analysis of these findings is hard to obtain with the limited data available. What appears to be a false positive prediction based on RegulonDB, may in fact be an unknown gene cluster, or one that is not well enough confirmed to appear in the database. Also a non-significant prediction, that is in fact a confirmed operon, does not mean that our p-values are too strict. Statistical significance by itself is simply not a necessary condition for a biological gene cluster.

## Conclusion and outlook

In this paper, we presented the first statistical model to estimate the significance of gene cluster predictions under an approximate common interval-based gene cluster model. The underlying p-value calculations integrate all parameters that influence the quality of gene cluster conservation. Namely, the number of genomes in which the gene cluster is detected, the size of the gene cluster, the degree of conservation within the different occurrences, as well as the genome-wide frequencies of the genes involved. To keep the computation time feasible, we had to make some simplifying assumptions in the p-value calculation, but we have experimentally shown that our estimations are remarkably close to empirically derived p-values. An analysis of a set of well annotated genomes has proven that our method is able to re-discover known, highly conserved gene clusters with p-values clearly showing that such conservation did not occur by chance. The gene cluster at position 20 in our output list (functional annotation: arginine biosynthesis) still receives a highly significant p-value of 5.20 · 10^−177^. We also studied clusters with low significance and observed known operons with p-values below the significance threshold of 0.05, as well as unknown clusters with significant p-values. However, due to the limited data available it was not possible to distinguish between false and true positives.

Although the simplifying assumptions seem to have little effect in practice, it would be an interesting next step to study more accurate models in the future. In particular, the assumption that the probabilities of observing a cluster are statistically independent in overlapping intervals could be omitted. This could be achieved by accounting for such dependencies in our calculations, for example by employing the inclusion/exclusion principle. Alternatively our present p-value approximation might be amenable to control by the Chen-Stein method [37].

Another strong assumption in our model is that any two genomes show fully randomized gene order, unless evolutionary pressure prohibits it. This assumption may be violated if we analyze genomes of closely related species or strains. In this case, significances will be overrated by our p-value estimation. For the dataset analyzed in this study at least, this problem is less severe than one might expect. Even strains of the same species show a relatively high amount of random shuffling (Additional file 1). Nevertheless, by lowering the quorum parameter to $k^{\prime }=\text{5}$, we observe that many conserved regions are detected, where all or most species are *Mycobacteria*. In cases where more closely related strains are analyzed, we suggest several workarounds in Additional file 1. In the future, it will be an interesting problem to include incomplete randomization into our statistical model.

## Competing interests

The authors declare that they have no competing interests.

## Supplementary Material

Additional file 1 **(PDF)**. Click here for file
