# Supplementary material for: Statistics for approximate gene clusters
Source: BMC Bioinformatics. 2013 Dec 13;14(Suppl 15):S14. doi: 10.1186/1471-2105-14-S15-S14 (PMC3908651; doi:10.1186/1471-2105-14-S15-S14)
Supplement: Additional file 1 — (PDF). [file 1471-2105-14-S15-S14-S1.PDF]

Supplementary Material

Log-log plot of number of genes with a particular gene family size

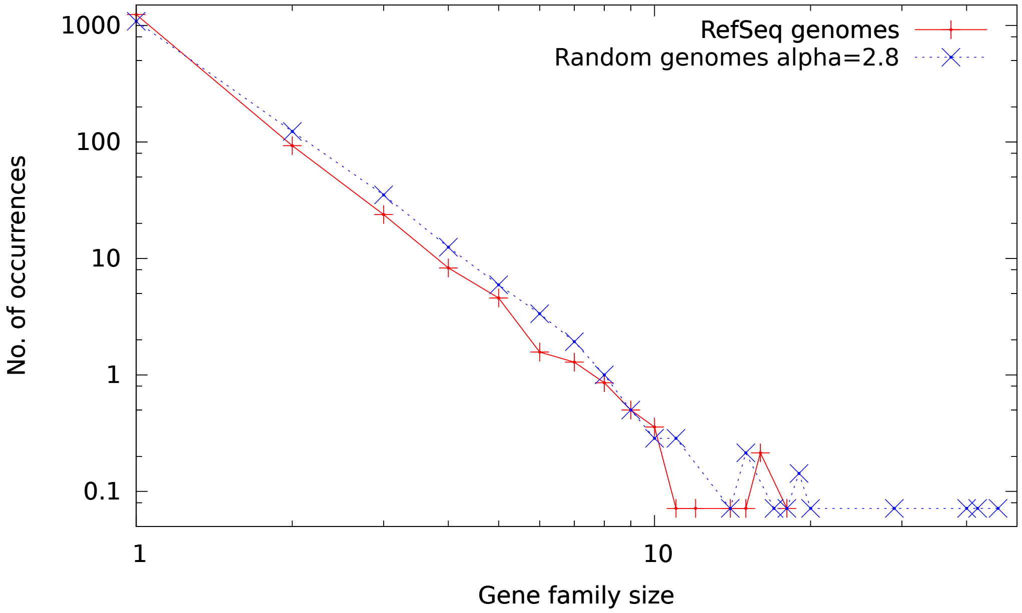

Supplementary Fig. 1. Log-log plot of number of genes with a particular gene family size. 14 biological genomes with 1250 to 1750 genes from Suppl. Table 1 (solid line); random genomes drawn using the Pareto distribution with $\alpha = 2.8$ and identical length distribution (dashed line).

List of genomes used in our evaluation

Supplementary Table 1. Dataset of 119 genomes used in experimental evaluation. “refSeq” is the reference number (add prefix ‘NC_’) from the RefSeq database, see Pruitt *et al.*, *Nucleic Acids Res.*, 40:D130–135, 2012. “# G” is the number of genes in the genome.

Species name	refSeq	# G
Aeropyrum pernix K1	000854	1700
Aquifex aeolicus VF5	000918	1529
Archaeoglobus fulgidus DSM 4304	000917	2420
Bacillus anthracis str. Ames	003997	5311
Bacillus halodurans C125	002570	4066
Bacillus subtilis subsp. subtilis str. 168	000964	4105
Bacteroides thetaiotaomicron VPI5482	004663	4778
Bdellovibrio bacteriovorus HD100	005363	3587
Bordetella bronchiseptica RB50	002927	4994
Bordetella parapertussis 12822	002928	4185
Bordetella pertussis Tohama I	002929	3436
Borrelia burgdorferi B31	001318	851
Bradyrhizobium japonicum USDA 110	004463	8317
Buchnera aphidicola str. APS (Acyrthosiphon pisum)	002528	564
Buchnera aphidicola str. Bp (Baizongia pistaciae)	004545	504
Buchnera aphidicola str. Sg (Schizaphis graminum)	004061	546
Candidatus Blochmannia floridanus	005061	583
Caulobacter crescentus CB15	002696	3737
Chlamydia muridarum Nigg	002620	904
Chlamydophila caviae GPIC	003361	998
Chlamydophila pneumoniae AR39	002179	1112
Chlamydophila pneumoniae CWL029	000922	1052
Chlamydophila pneumoniae J138	002491	1069
Chlamydophila pneumoniae TW183	005043	1113
Chlorobium tepidum TLS	002932	2252
Chromobacterium violaceum ATCC 12472	005085	4407
Clostridium acetobutylicum ATCC 824	003030	3672
Clostridium tetani E88	004557	2373
Corynebacterium diphtheriae NCTC 13129	002935	2272
Corynebacterium efficiens YS314	004369	2950
Corynebacterium glutamicum ATCC 13032	006958	3057
Dehalococcoides ethenogenes 195	002936	1580
Enterococcus faecalis V583	004668	3113
Escherichia coli O157H7 str. Sakai	002695	5230
Escherichia coli str. K12 substr. MG1655	000913	4132
Fusobacterium nucleatum subsp. nucleatum ATCC 25586	003454	2067
Geobacter sulfurreducens PCA	002939	3446
Gloeobacter violaceus PCC 7421	005125	4430
Haemophilus ducreyi 35000HP	002940	1717
Haemophilus influenzae Rd KW20	000907	1657
Helicobacter hepaticus ATCC 51449	004917	1875
Helicobacter pylori 26695	000915	1576
Helicobacter pylori J99	000921	1489
Lactobacillus johnsonii NCC 533	005362	1821
Lactobacillus plantarum WCFS1	004567	3007
Lactococcus lactis subsp. lactis Il1403	002662	2321
Listeria innocua Clip11262	003212	2968
Listeria monocytogenes EGDe	003210	2846
Listeria monocytogenes str. 4b F2365	002973	2821
Mesorhizobium loti MAFF303099	002678	6743
Methanopyrus kandleri AV19	003551	1687
Methanosarcina acetivorans C2A	003552	4540
Methanosarcina mazei Go1	003901	3370
Methanothermobacter thermautotrophicus str. Delta H	000916	1873
Mycobacterium avium subsp. paratuberculosis K10	002944	4350
Mycobacterium leprae TN	002677	1605
Mycobacterium tuberculosis CDC1551	002755	4189
Mycobacterium tuberculosis H37Rv	000962	3989
Mycoplasma gallisepticum R	004829	726
Mycoplasma genitalium G37	000908	477
Mycoplasma mobile 163K	006908	633
Mycoplasma mycoides subsp. mycoides SC str. PG1	005364	1016
Mycoplasma penetrans HF2	004432	1037
Mycoplasma pneumoniae M129	000912	689
Mycoplasma pulmonis UAB CTIP	002771	782
Nanoarchaeum equitans Kin4M	005213	536
Neisseria meningitidis MC58	003112	2063
Neisseria meningitidis Z2491	003116	2049
Nitrosomonas europaea ATCC 19718	004757	2461
Oceanobacillus iheyensis HTE831	004193	3500
Onion yellows phytoplasma OYM	005303	754
Pasteurella multocida subsp. multocida str. Pm70	002663	2015
Photorhabdus luminescens subsp. laumondii TTO1	005126	4683
Prochlorococcus marinus str. MIT 9313	005071	2269
Prochlorococcus marinus subsp. marinus str. CCMP1375	005042	1883
Prochlorococcus marinus subsp. pastoris str. CCMP1986	005072	1717
Pseudomonas putida KT2440	002947	5350
Pseudomonas syringae pv. tomato str. DC3000	004578	5475
Pyrobaculum aerophilum str. IM2	003364	2605
Pyrococcus abyssi GE5	000868	1896
Pyrococcus furiosus DSM 3638	003413	2125
Pyrococcus horikoshii OT3	000961	1955
Ralstonia solanacearum GMI1000	003295	3440
Rickettsia conorii str. Malish 7	003103	1374
Rickettsia prowazekii str. Madrid E	000963	835
Salmonella enterica subsp. enterica serovar Typhi Ty2	004631	4318
Salmonella typhimurium LT2	003197	4425
Shewanella oneidensis MR1	004347	4318
Shigella flexneri 2a str. 2457T	004741	4061
Shigella flexneri 2a str. 301	004337	4177
Sinorhizobium meliloti 1021	003047	3341
Solibacter usitatus Ellin6076	008536	7826
Staphylococcus aureus subsp. aureus MW2	003923	2632
Streptococcus agalactiae 2603V/R	004116	2124
Streptococcus agalactiae NEM316	004368	2094
Streptococcus mutans UA159	004350	1960
Streptococcus pneumoniae R6	003098	2043
Streptococcus pneumoniae TIGR4	003028	2105
Streptococcus pyogenes M1 GAS	002737	1697
Streptococcus pyogenes MGAS315	004070	1865
Streptococcus pyogenes MGAS8232	003485	1839
Streptococcus pyogenes SSI1	004606	1861
Streptomyces avermitilis MA4680	003155	7580
Streptomyces coelicolor A3(2)	003888	7769
Sulfolobus solfataricus P2	002754	2977
Sulfolobus tokodaii str. 7	003106	2825
Synechococcus sp. WH 8102	005070	2519
Thermoanaerobacter tengcongensis MB4	003869	2588
Thermoplasma acidophilum DSM 1728	002578	1482
Thermoplasma volcanium GSS1	002689	1499
Thermosynechococcus elongatus BP1	004113	2476
Thermotoga maritima MSB8	000853	1858
Treponema denticola ATCC 35405	002967	2767
Tropheryma whipplei TW08/27	004551	783
Tropheryma whipplei str. Twist	004572	808
Wolinella succinogenes DSM 1740	005090	2042
Xanthomonas axonopodis pv. citri str. 306	003919	4312
Xylella fastidiosa 9a5c	002488	2766
Yersinia pestis CO92	003143	3885

Complete list of gene clusters with p-values

Supplementary Table 2. All gene clusters of searching *Mycobacterium tuberculosis* CDC1551 against 118 bacterial genomes. Clusters sorted by p-values, computed using the "individual distance bounds" method. "G" is the number of different genes in the reference gene cluster; "GN" is the number of genomes where the reference gene cluster is actually found. "Distance to ref." is the observed distances between the reference gene cluster and its occurrences. The "p-score" is the negative $\log_{10}$ of the p-value, before and after FDR correction. The description contains either the names of contained genes or a function extracted from the gene annotations.

			distance to ref.				corr.	
ID	G	GN	min	max	avg	p-score	p-score	description
1	9	108	2	5	2.8	1314.43	1307.49	30S/50S ribosomal subunit
2	7	114	0	3	1.6	1258.12	1251.18	30S/50S, rpoA, infA
3	6	91	0	2	0.7	1031.47	1024.83	ATP synthase
4	9	57	0	5	1.4	896.31	890.57	NADH dehydrogenase
5	8	108	3	5	4.1	716.68	711.29	30S/50S ribosomal subunit
6	8	88	0	5	4.2	569.88	564.63	phosphate ABC transporter
7	8	93	0	5	4.1	486.80	481.67	infB, rfbA, nusA, hypothetical protein
8	8	79	3	5	4.6	367.33	362.27	putative/peptide ABC transporter
9	8	62	3	5	4.4	294.41	289.40	sugar ABC transporter
10	8	65	2	5	4.1	290.24	285.24	N-acetylmuramoyl, cell division
11	4	33	0	0	0.0	272.99	267.55	succinate dehydrogenase
12	8	51	3	5	4.9	221.73	216.79	pdhA/B/C
13	8	48	2	5	4.9	216.54	211.62	ATP-dependent (Clp) protease, trigger factor
14	8	58	0	5	4.2	216.12	211.20	50S L31, prfA, thrA/B/C, rho, hemK
15	8	50	4	5	4.9	213.61	208.70	hisA/C/F/H
16	6	32	0	2	1.7	200.11	194.80	dnaA/N, gyrA/B, recF
17	6	27	1	2	1.7	194.39	189.10	carA/B, pyrC/B/R
18	8	67	4	5	5.0	192.56	187.69	elongation factor Tu, G; 30S S7
19	8	29	4	5	4.5	190.62	185.75	sulfate ABC transporter
20	8	44	2	5	4.3	181.13	176.28	argB/C/D/G/H/F/R
21	8	44	2	5	4.8	165.08	160.26	DNA polymerase III, recR, hypothetical protein
22	8	39	3	5	4.9	164.93	160.11	ilvB/C/H
23	6	22	2	2	2.0	164.78	159.53	kdpB/C/D/E
24	4	18	0	0	0.0	151.58	146.21	nitrate reductase
25	8	34	3	5	4.5	142.14	137.36	folB/E/K/P, ftsH
26	8	30	2	5	4.8	138.88	134.11	putative ABC transporter
27	8	31	3	5	4.5	114.80	110.06	parA/B, gidB
28	8	27	2	5	4.5	112.25	107.51	ruvA/B/C
29	8	31	2	5	4.7	104.10	99.38	goeES/EL, gcp, rimI
30	8	23	4	5	4.9	99.26	94.55	gmk, rpoZ, dfp
31	4	13	0	0	0.0	91.64	86.32	thiE/G/O/S
32	6	11	0	1	0.4	91.49	86.30	hemL, ccsA/B
33	8	27	2	5	4.8	90.28	85.58	adk, map, secY
34	8	22	5	5	5.0	79.57	74.89	rplY, prsA, ispE
35	8	35	4	5	4.5	78.65	73.98	putative ABC transporter
36	7	14	1	3	2.7	77.79	72.74	nrdE/F/H/I
37	4	10	0	0	0.0	74.76	69.47	—
38	4	11	0	0	0.0	73.46	68.18	qcrA/B/C, ctaE
39	8	21	0	5	4.4	72.58	67.92	cydA/B/C/D
40	7	11	3	3	3.0	71.64	66.61	bioA/B/D/F
41	8	26	1	5	4.6	70.20	65.56	rpe, sunL, priA, fmt
42	8	17	5	5	5.0	69.61	64.97	purF/L/M/Q
43	8	17	1	5	4.6	69.25	64.61	rpsT, comEA/EC, holA
44	8	24	2	5	4.4	60.93	56.32	fabG, atoA/B/D
45	7	11	1	3	2.4	59.66	54.67	aroB/D/K/Q, efp, nusB, pepQ
46	8	17	2	5	4.3	56.87	52.27	phoH, era, recO
47	8	20	2	5	4.7	56.25	51.65	—
48	8	17	2	5	4.3	53.76	49.17	smpB, ftsE/X, prfA/B
49	8	15	1	5	4.4	46.85	42.28	smc, rnc, ftsY, fpg
50	8	12	4	5	4.8	40.95	36.39	rnhB, hypothetical proteins
51	8	13	4	5	4.7	35.78	31.24	rfbD/E, glaxocyl transferase, quinone oxidoreductase
52	8	10	2	5	4.6	35.18	30.63	treX/Y/Z, glgX/Y/Z
53	8	11	0	5	3.9	32.39	27.86	murI, cysM, rph, hypothetical proteins
54	8	16	3	5	4.5	32.22	27.69	mtrA/B, secA, tmk
55	8	10	3	5	4.1	29.85	25.33	kasA, fabD/F, aceE, acpP
56	8	10	3	5	4.3	29.84	25.33	metG, ksgA, hypothetical proteins
57	8	11	2	5	4.4	29.33	24.82	pknB, pbpA, pabA
58	8	11	2	5	4.2	28.02	23.51	cobI/J/K/L/M
59	8	10	2	5	3.2	27.62	23.12	pyrG, ppnK, xerD, recN, tlyA
60	8	12	1	5	4.3	27.17	22.67	pgsA, ftsK, thyX, dapA
61	8	10	0	5	3.1	25.35	20.85	eno, mdf, hypothetical proteins
62	8	12	4	5	4.9	24.86	20.37	enoyl-CoA hydratase, acyl-CoA synthetase/dehydrogenase
63	8	10	2	5	4.2	15.12	10.66	htrA, sigE, ABC transporter

Supplementary Table 3. All gene clusters of searching *Mycobacterium tuberculosis* CDC1551 against 118 bacterial genomes. Clusters sorted by p-values, calculated using the “global distance bound” method. This Table shows different occurrences of mostly the same clusters as in Supplementary Table 2 as only the best scoring one is reported. “G” is the number of different genes in the reference gene cluster; “GN” is the number of genomes where the reference gene cluster is actually found. “Distance to ref.” is the observed distances between the reference gene cluster and its occurrences. The “p-score” is the negative $\log_{10}$ of the p-value, before and after FDR correction. The description contains either the names of contained genes or a function extracted from the gene annotations.

			distance to ref.				corr.	
ID	G	GN	min	max	avg	p-score	p-score	description
1	9	66	2	2	2.0	952.87	945.93	30S/50S ribosomal subunit
2	5	112	0	1	0.5	879.37	872.42	30S/50S, rpoA
3	6	76	0	1	0.4	769.67	763.42	ATP synthase
4	6	93	2	2	2.0	701.24	695.41	30S/50S ribosomal subunit
5	9	47	0	2	0.8	686.92	681.12	NADH dehydrogenase
6	4	68	0	0	0.0	560.11	554.12	phosphate ABC transporter
7	5	57	0	1	0.8	443.65	438.07	infB, rfbA, nusA
8	8	79	3	5	4.6	304.16	299.07	putative/peptide ABC transporter
9	4	33	0	0	0.0	272.99	267.55	succinate dehydrogenase
10	7	42	2	3	2.8	270.78	265.51	N-acetylmuramoyl, cell division
11	8	62	3	5	4.4	233.84	228.84	sugar ABC transporter
12	8	51	3	5	4.9	219.07	214.10	pdhA/B/C
13	8	48	2	5	4.9	214.77	209.81	ATP-dependent (Clp) protease, trigger factor
14	8	50	4	5	4.9	209.23	204.29	hisA/C/F/H
15	8	58	0	5	4.2	209.01	204.07	50S L31, prfA, thrA/B/C, rho, hemK
16	6	32	0	2	1.7	199.36	194.05	dnaAN, gyrAB, recF
17	8	67	4	5	5.0	190.91	186.03	elongation factor Tu, G; 30S S7
18	6	27	1	2	1.7	190.80	185.51	carA/B, pyrC/B/R
19	5	23	0	1	0.8	174.03	168.71	cell division, conserved hypothetical proteins
20	8	44	2	5	4.3	171.92	167.06	argB/C/D/G/H/F/R
21	6	22	2	2	2.0	164.78	159.52	kdpB/C/D/E
22	8	39	3	5	4.9	162.38	157.55	ilvB/C/H
23	8	44	2	5	4.8	161.73	156.90	DNA polymerase III, recR
24	4	18	0	0	0.0	151.58	146.21	nitrate reductase
25	8	29	4	5	4.5	151.54	146.73	sulfate ABC transporter
26	8	34	3	5	4.5	137.36	132.58	folB/E/K/P, ftsH
27	8	30	2	5	4.8	135.95	131.16	glycine betaine/carnitine/choline ABC transporter
28	8	31	3	5	4.5	113.22	108.46	parA/B, gidB
29	8	27	2	5	4.5	107.33	102.59	ruvA/B/C
30	8	31	2	5	4.7	102.34	97.62	goeES/EL, gcp, rimI
31	8	23	4	5	4.9	97.90	93.19	gmk, rpoZ, dfp
32	4	13	0	0	0.0	91.64	86.32	thiE/G/O/S
33	8	27	2	5	4.8	89.20	84.50	adk, map, secY
34	6	11	0	1	0.4	88.51	83.33	hemL, ccsA/B
35	8	22	5	5	5.0	79.57	74.89	rplY, prsA, ispE
36	7	14	1	3	2.7	75.06	70.01	nrdE/F/H/I
37	4	11	0	0	0.0	73.46	68.18	qcrA/B/C, ctaE
38	7	11	3	3	3.0	71.64	66.61	bioA/B/D/F
39	9	19	4	5	4.9	71.03	66.37	putative ABC transporter
40	8	21	0	5	4.4	69.97	65.32	cydA/B/C/D
41	8	17	5	5	5.0	69.61	64.97	purF/L/M/Q
42	8	26	1	5	4.6	69.37	64.73	rpe, sunL, priA, fmt
43	8	17	1	5	4.6	68.29	63.66	rpsT, comEA/EC, holA
44	7	11	1	3	2.4	59.46	54.46	aroB/D/K/Q, efp, nusB, pepQ
45	8	17	2	5	4.3	55.80	51.20	phoH, era, recO
46	8	20	2	5	4.7	55.03	50.20	—
47	8	24	2	5	4.4	53.21	48.62	fabG, atoA/B/D
48	8	17	2	5	4.3	52.21	47.62	smpB, ftsE/X, prfA/B
49	8	15	1	5	4.4	46.44	41.86	smc, rnc, ftsY, fpg
50	8	12	4	5	4.8	40.82	36.26	rnhB, hypothetical proteins
51	8	13	4	5	4.7	35.03	30.48	rfbD/E, glaxocyl transferase, quinone oxidoreductase
52	8	10	2	5	4.6	33.62	29.08	treX/Y/Z, glgX/Y/Z
53	8	16	3	5	4.5	31.94	27.40	mtrA/B, secA, tmk
54	8	11	0	5	3.9	31.87	27.34	murI, cysM, rph, hypothetical proteins
55	8	10	3	5	4.3	29.58	25.05	metG, ksgA
56	8	10	3	5	4.1	28.94	24.42	kasA, fabD/F, aceE, acpP
57	8	11	2	5	4.4	27.83	23.32	pknB, pbpA, pabA
58	8	10	2	5	3.2	27.00	22.50	pyrG, ppnK, xerD, recN, tlyA
59	8	11	2	5	4.2	26.02	21.51	cobI/J/K/L/M
60	8	12	1	5	4.3	25.33	20.83	pgsA, ftsK, thyX, dapA
61	8	10	0	5	3.1	23.73	19.24	eno, mdf, hypothetical proteins
62	8	12	4	5	4.9	23.14	18.65	enoyl-CoA hydratase, acyl-CoA synthetase/dehydrogenase
63	8	11	3	5	4.5	15.17	10.70	gpm, senX3, regX3, glycosyl transferase
64	8	10	2	5	4.2	14.34	9.89	htrA, sigE, ABC transporter

Gene cluster predictions with low significance

Supplementary Table 4. Gene cluster predictions with high p-values detected for parameter settings ($s = 3, \delta = 1, k' = 9$), ($s = 4, \delta = 4, k' = 8$) and ($s = 6, \delta = 7, k' = 7$). For each setting all predictions with a p-value > 0.05 (corrected p-score $<$ -1.3), and the same number of predictions with the biggest p-values < 0.05 in each setting are listed. Results are sorted by corrected p-value. Overlaps with E. coli operons obtained from the RegulonDB database are highlighted in green. The list is continued in Suppl. Table 5.

G	GN	distance to ref. min	max	avg	p-score	corr. p-score	description
7	14	2	7	4.5	4.62	1.76	yifEB, ilvLMEDA
9	11	1	7	4.1	4.53	1.67	glnH, dps, rhtA, ompX, ybiPRS, yliL, mntR
8	13	3	7	5.1	4.34	1.48	ynfM, asr, ydgUD, mdtIJ, tqsA, pntBA
9	19	0	7	5.7	4.31	1.45	nudB, aspS, yecDE, yecN, cmoAB, torZY, cutC
9	14	3	7	6.2	4.22	1.36	glcED, glcC, insH, yghQ, yghRS, yghTU, pitB, gsp, hybG
6	10	0	7	2.8	4.22	1.36	yccFTU, helD, mgsA, hspQ
8	10	2	7	4.8	3.90	1.04	mdoH, yceKA, yceIJ, msyB, mdtG, lpxL
9	12	4	7	6.1	3.80	0.95	yliEF, yliGIJ, bssR, dacC, deoR, ybjG
9	12	4	7	6.4	3.56	0.71	rhaAB, rhaSR, rhaT, sodA, kdgT, yiiM, cpxA
8	15	4	7	5.6	3.55	0.70	ydcWXYZ, yncLABC
4	9	2	2	2.0	3.58	0.34	yjhHI, sgcRE
3	12	0	2	1.1	3.50	0.26	ynbABC
7	11	2	6	3.8	3.02	0.18	trmH, recG, gltS, yicEH, yicIJ
6	14	0	7	2.8	2.90	0.06	htpG, adk, hemH, aes, gsk, ybaL
9	14	0	7	4.5	2.90	0.05	gutMQ, srlR, norR, norVW, hypF, hydN, ascG
3	9	0	2	1.3	3.15	-0.09	ybdGF, nfsB
4	11	2	4	3.3	2.71	-0.22	rspA, ynfA, ynfB, speG
5	9	2	4	3.3	2.68	-0.25	ybdGFJK, nfsB
3	10	0	2	0.3	2.98	-0.25	cytR, priA, rpmE
3	9	1	2	1.6	2.93	-0.30	uvrY, yecF, sdiA
3	9	0	2	0.5	2.85	-0.38	ybiRST
3	9	0	2	1.6	2.82	-0.41	ygeWXY
3	9	0	2	0.6	2.81	-0.42	aes, gsk, ybaL
6	8	4	4	4.0	2.45	-0.48	ydcKLMO, tehAB
4	9	0	2	0.8	2.72	-0.51	ytjC, rob, creAB
3	9	1	2	1.3	2.66	-0.57	ydcK, tehAB
5	9	2	4	2.4	2.31	-0.61	rfaYJIBSPG
6	11	2	7	4.3	2.18	-0.65	sohA, yhaV, agaRS, agaV, kbaZ
5	9	2	4	2.9	2.24	-0.68	eamA, ydeEHI, yneM
8	30	0	7	5.1	2.01	-0.82	yigIM, yigL, pldB, pldA, recQ, rhtCB, metR
9	14	2	7	5.8	2.01	-0.83	ygjGHI, ygjJK, ebgR, ebgAC, fadH
4	14	2	4	2.6	2.04	-0.88	mrcA, nudE, yrfFG
6	18	2	7	4.8	1.93	-0.90	eptB, yhjXY, tag, yiaC, bisC
4	9	2	2	2.0	2.24	-0.99	lacZ, lacI, mhpRA
5	8	2	4	2.6	1.88	-1.04	bisC, yiaDFG, ghrB
5	13	2	4	2.8	1.88	-1.04	ycfQST, bhsA, mfd
3	10	0	2	0.9	2.16	-1.07	gudP, yqcA, truC
5	10	2	4	2.7	1.78	-1.13	yhhWXY, yhhZ, insA
4	10	0	3	1.4	1.79	-1.13	rdoA, dsbA, yihFG
6	14	0	4	3.3	1.74	-1.18	gadA, yhjABCD, treF
9	11	3	7	6.2	1.61	-1.21	insI, ydbCD, ynbABCD, azoR, hrpA
6	14	2	7	3.7	1.60	-1.23	sirA, yhhQST, dcrB, acpT

Supplementary Table 5. Continues Suppl. Table 4

G	GN	distance to ref. min	max	avg	p-score	corr. p-score	description
6	19	1	7	4.0	1.45	-1.37	fecDCBA, fecRI, insA
4	10	0	2	1.2	1.84	-1.38	ybaEO, cof, mdlAB
8	10	4	7	5.6	1.15	-1.67	pheP, ybdGFJK, nfsB, hokE, insL
7	17	3	7	5.1	1.09	-1.72	tdcAR, yhaBC, garKRL
4	12	0	2	1.4	1.41	-1.81	treF, yhjBCD
3	10	0	2	0.9	1.12	-2.10	tas, lplT, aas
5	9	1	4	2.3	0.78	-2.12	ygfZF, yqfAB, bglA
3	15	0	2	1.0	1.03	-2.19	yfiCE, srmB
6	11	2	7	3.7	0.59	-2.22	pyrC, yceBH, grxB, mdtH, rimJ
6	17	0	7	3.6	0.57	-2.24	yihQR, yihSTU, yihV
5	8	0	4	2.4	0.67	-2.24	ydaLMN, dbpA, ttcA
7	11	4	7	5.2	0.53	-2.28	yfdRST, torI, dsdC, dsdXA
5	9	3	4	3.1	0.58	-2.32	rcsA, dsrB, yodD, yedPQ
6	10	0	7	2.8	0.42	-2.39	sugE, blc, ampC, frdDCB
5	11	2	4	3.1	0.50	-2.40	eptB, yhjXY, tag, yiaC
3	11	0	2	0.8	0.81	-2.41	mtgA, elbB, arcB
5	14	0	4	2.5	0.49	-2.42	ygdQR, tas, lplT, aas
6	9	2	4	3.5	0.46	-2.44	ygiSTVW, mqsR, qseB
5	9	0	4	1.9	0.46	-2.45	yfcCDEFG, folX
7	11	5	7	6.2	0.20	-2.60	rspA, ynfACDE, ynfB, speG
8	13	0	7	3.6	0.14	-2.66	hupB, ppiD, ybaVWEO, queC, cof
6	26	0	7	4.0	0.13	-2.68	secA, mutT, yacGF, coaE, guaC
7	12	1	7	3.5	0.10	-2.70	rimI, yjjG, yjjUV, prfC, osmY, ytjA
7	14	2	7	5.8	-0.13	-2.92	hyuA, yqeABC, ygfJK, ssnA
6	13	2	7	3.9	-0.12	-2.92	mrr, yjiAX, yjiY, tsr, yjjL
4	9	0	4	1.6	-0.08	-2.98	uspG, ybdR, rnk, rna
3	10	0	2	1.0	0.23	-2.99	yieP, hdfR, yifE
7	10	5	7	5.7	-0.21	-3.01	yjhP, yjiQX, yjiS, nanMC, fimBE
3	9	0	2	0.8	0.19	-3.03	tdcCBA
3	10	0	2	1.1	0.18	-3.04	ycaCDM
4	9	2	3	2.5	-0.21	-3.11	uxuR, yjiCDE
6	15	0	7	3.5	-0.38	-3.18	yihFGAI, polA, hemN
4	12	0	4	2.1	-0.37	-3.27	dmsC, ycaCDM
6	10	1	7	3.6	-0.55	-3.34	yidLPEQR, ibpBA
3	9	0	2	0.8	-0.14	-3.36	tsr, yjjLM
3	13	0	2	1.0	-0.33	-3.55	rutBA, rutR
3	10	0	2	1.3	-0.39	-3.60	ycjZ, mppA, ynaI
8	10	6	7	6.2	-0.93	-3.71	insA, yagJK, yagLM, yagNP, intF
3	9	1	2	1.4	-0.56	-3.77	yohDF, dusC
4	11	0	4	2.1	-0.90	-3.79	ybhADH, pgl
6	13	3	7	4.6	-1.03	-3.81	aidB, yjfNOP, ulaRG
6	13	0	7	4.5	-1.34	-4.12	ydaLMN, dbpA, ttcA, intR

Conservation pattern of the dnaA-dnaN-recF-gyrB-gyrA gene cluster

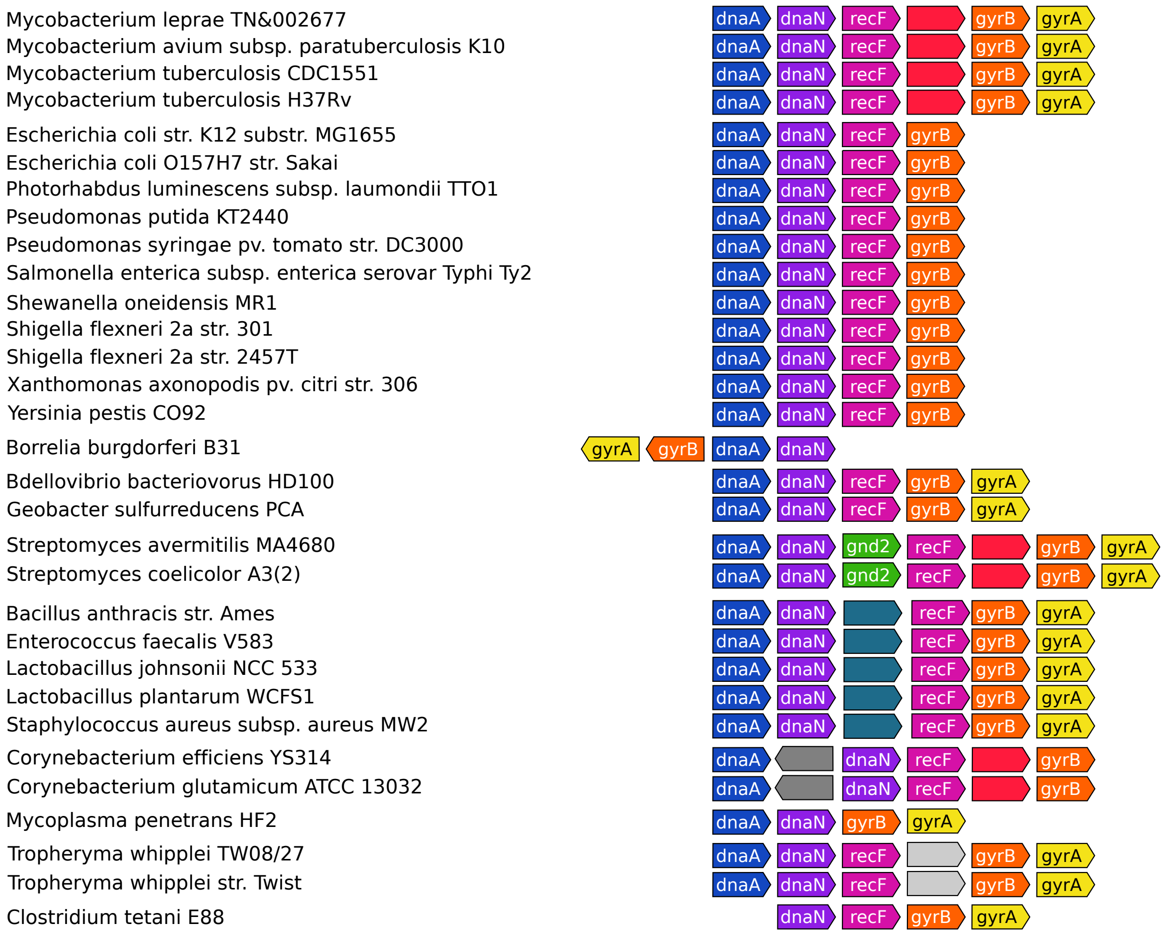

Supplementary Fig. 2. Detailed conservation pattern for a gene cluster detected in a dataset of 118 bacterial genomes using *Mycobacterium tuberculosis* as reference genome and parameters $\delta = 2$, $s = 6$. The gene cluster occurs in 31 genomes and has a corrected p-score of 194.05 which strongly suggests that it is not a chance occurrence. In fact, this is a known gene cluster that is involved in genome replication. The core genes of the cluster are dnaA, dnaN, recF, gyrA and gyrB. The variants in which the cluster occurs are shown above. The contained genes are either labeled with their name, or left blank in case of hypothetical genes.

Evaluation of strain similarities

If we include several strains from one species, or possibly also closely related species, this interferes with our assumption of complete randomization of gene order. This leads to problems both with respect to the quorum parameter and our statistical evaluation. Regarding the quorum parameter, any gene cluster found in one strain of a species is likely found also in all other strains. In the worst case, (almost) everything is a gene cluster. This problem may result in increased running times, as many potential gene clusters have to be evaluated; but these bogus gene clusters can be discarded by a sound statistical evaluation.

In our statistical evaluation, we compare against the null model that gene order is random, and this may be violated for strains of the same species which may show identical gene order in large parts. To this end, we wanted to evaluate how pressing this problem is, for the dataset at hand. A straightforward measure to evaluate how strongly gene order is conserved, is the breakpoint distance. For a normalized breakpoint distance of 1 we have no conserved gene adjacencies between two genomes, whereas a normalized breakpoint distance of 0 corresponds to identical gene order. Unfortunately, the breakpoint distance is defined only for permutations, whereas the genomes used here are are sequences, as any gene number can occur multiple times.

To this end, we use a simple and straightforward generalization of the breakpoint distance to strings: Let S_1, S_2 be the genomes over the alphabet Σ. For each genome S_i we build a vector $v^i \in \mathbb{N}^m$ with $m := \binom{|\Sigma|+1}{2}$, where each entry counts the number of one unordered gene pair $x, y \in \Sigma$. We define the normalized distance between S_1 and S_2 as

$$d(S_1, S_2) := \frac{\sum_{l=0}^{m} \left|v_l^1 - v_l^2\right|}{|S_1| - 1 + |S_2| - 1}.$$

Again, this distance is 1 if there are no conserved gene adjacencies, and 0 for identical strings.

Figure 3 shows a heat map plot of the resulting distances for the 119 genomes from Suppl. Table 1. For some closely related species (especially several *E. coli* species in the bottom left corner of the plot, and *Streptococcus* species in the upper right corner) the distances are relatively small. But for most genome pairs, distances are very high and often above 0.9. We argue that for this dataset, computed p-values will not be significantly distorted by the small amount of closely related genomes.

We suggest the following workaround to the problem for datasets containing more similar genomes: In case we know in advance whether two genomes belong to strains of the same species, we can flag these strains accordingly. From every family of strains, at most one should contribute to p-value calculation and quorum. In case no information about strains is available, a pairwise comparison of genome order as indicated above allows us to find strains or "too closely related" genomes. But even when strains are not explicitly flagged as such, the statistical evaluation will work as expected; the only restriction is that possibly, gene clusters across stains get a better p-value than they should.

Implementation details

The computation of reference gene clusters as well as the calculation of p-values is implemented in C/C++. We noticed that the smallest p-values occurring in our calculations are lower than the smallest value, greater than zero, that can be stored by a double precision data type, which is about 10^{-308}. To avoid such low p-values being rounded down to zero we designed a custom data type, consisting of a floating point value as significant and an

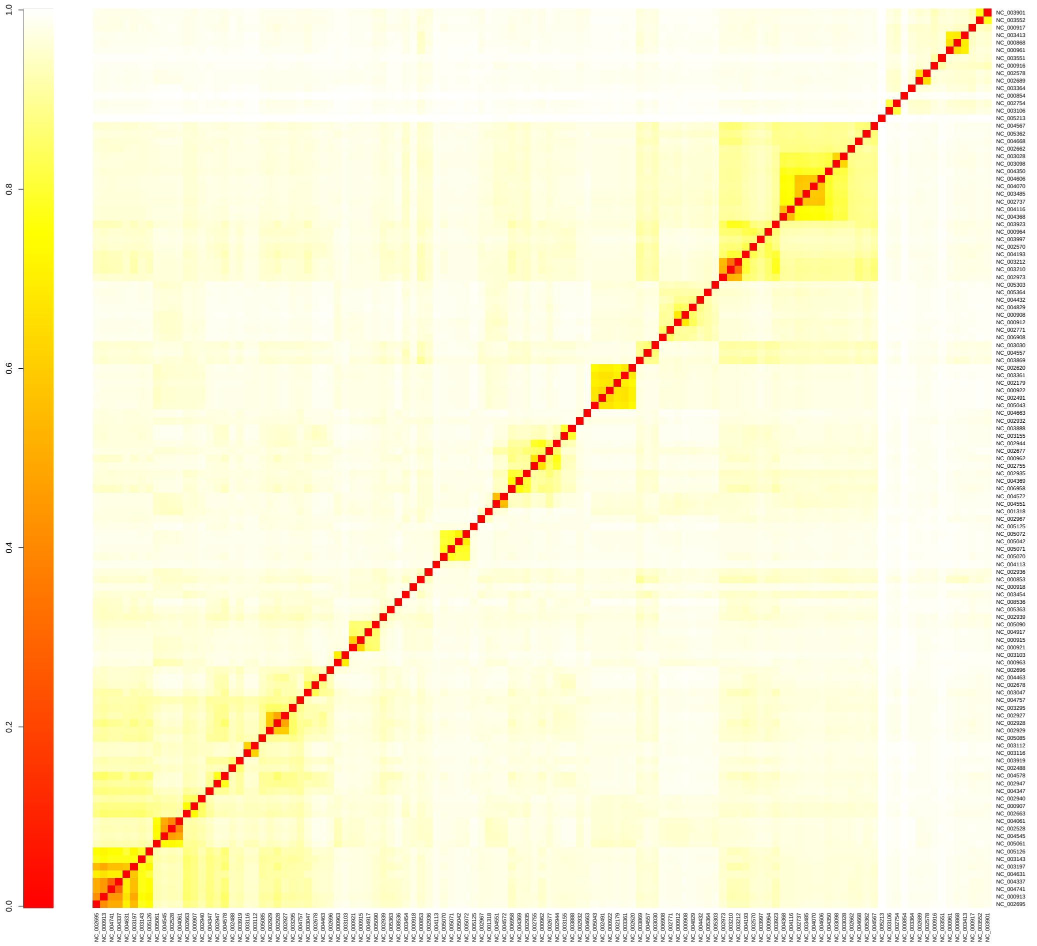

Supplementary Fig. 3. Heat map plot of genome distances computed by a simplified breakpoint distance between strings. This plot uses the 119 genomes from Suppl. Table 1, see there for species names. Genomes are ordered in the plot based on the phylogenetic tree by Ciccarelli *et al.*, *Science*, 311(5765):1283–1287, 2006.

integer exponent. Using this data type decreases the speed of our calculation. Therefore, it is only used to compute the significance of a gene cluster in multiple genomes (Section 4), where extremely small numbers arise from multiplying genome-wise p-values.
